# BetaH proteolysis unleashes an electrostatic-homing antibacterial polymorphic toxin

**DOI:** 10.1128/mbio.02804-25

**Published:** 2026-08-05

**Authors:** Daniel Mwangi, Maureen K. Thomason, Lauren M. Shull, Qing Tang, Joshua J. Woodward

**Affiliations:** 1 Department of Microbiology, University of Washington 312771, Seattle, Washington, USA; 2 Department of Biology, University of Texas at Arlington 12329, Arlington, Texas, USA; Biotechnical Faculty, University of Ljubljana, Ljubljana, Slovenia

**Keywords:** toxins, gram-positive bacteria, antagonism

## Abstract

Contact-dependent and diffusible proteinaceous polymorphic toxin systems (PTSs) mediate widespread bacterial competition. While bioinformatic analyses have identified diverse PTSs across bacterial phyla, experimental validation in gram-positive species remains limited. Here, we characterize a diffusible polymorphic toxin encoded by the *Staphylococcus aureus* S8-Ntox35 locus. We demonstrate that this system mediates inter-genus antagonism against *Listeria monocytogenes* via a bactericidal, heat-labile protein and that toxicity is enhanced following extracellular cleavage of the BetaH domain by the associated S8 peptidase. This processed toxin bears a peptide resembling a cationic antimicrobial peptide (CAMP) and facilitates intoxication of target cells by the Ntox35 RNase domain. Target cell resistance is impacted by known CAMP defense pathways, including DltABCD and MprF, and experimental evolution identified the ATP-binding cassette (ABC) transporter AnrAB as essential for intoxication. Unexpectedly, disruption of AnrAB abolished Ntox35 susceptibility, while simultaneously sensitizing cells to the proposed CAMP-like activity of the processed BetaH domain. These findings reveal a novel mechanism of inter-genus antagonism among Firmicutes (Bacillota) and establish a functional role for extracellular processing and ABC transporter-mediated susceptibility in PTS activity. Our work expands the known repertoire of diffusible toxins in gram-positive bacteria and sets the foundation for broader ecological and mechanistic investigation of the S8-associated polymorphic toxin system (S8-PTS).

**IMPORTANCE:**

Polymorphic toxin systems (PTSs) are widely used by bacteria to inhibit competitors, but diffusible proteinaceous toxins have been largely characterized in gram-negative species. Here, we mechanistically characterize a diffusible PTS in *Staphylococcus aureus* that mediates inter-genus antagonism in the Firmicutes phylum. This system employs a secreted S8 peptidase to cleave a BetaH-toxin fusion, releasing a previously caged cationic amphipathic α-helix that facilitates membrane targeting and intoxication of susceptible cells. We further show that toxin activity requires the target cell ATP-binding cassette (ABC) transporter AnrAB, revealing a novel route of entry and an evolutionary tradeoff between toxin susceptibility and antimicrobial peptide resistance. Together, these findings uncover a new mode of bacterial competition and highlight a broadly distributed toxin system with ecological relevance across Firmicutes.

## INTRODUCTION

To reproduce, bacteria must colonize environments where they can tolerate abiotic stresses and access nutrients needed for growth. Access to such niches is frequently challenged by genetically distinct organisms with overlapping ecological and nutritional requirements. Competition between these bacterial populations in both natural ecosystems and within mammalian hosts is therefore pervasive, driving the evolution of diverse antagonistic weaponry that restricts the replication of competitors and promotes clonal expansion (1).

Polymorphic toxin systems (PTSs) are a particularly prevalent molecular armament and can be broadly categorized into two functional types (2, 3). Contact-dependent systems utilize specialized secretion pathways and surface-associated proteins (e.g., type VI secretion system, type VII secretion system, and contact-dependent growth inhibition [CDI]) to deliver toxic effectors directly into neighboring cells (2–6). In contrast, contact-independent systems deploy diffusible antibacterial agents, including small molecules and protein toxins (e.g., colicins, umbrella particles, and extracellular contractile injection systems) that act at a distance (7–11).

Both classes of PTSs share a modular, multidomain architecture in which a toxin domain targeting essential cellular processes is coupled to domains responsible for targeting and/or delivery. This organization enables diversification through fusion of distinct toxic effectors to conserved delivery modules. Although toxin domains are highly variable and encompass a broad range of activities—including peptidoglycan degradation, membrane disruption, and nucleic acid cleavage—the conserved delivery components often restrict host range, typically limiting activity to closely related species (3, 12). To date, broad-acting diffusible antibacterial proteins remain rare, and such systems have not been previously reported in Firmicutes, recently renamed Bacillota.

Recent bioinformatic approaches integrating domain architecture and genomic context have accelerated the discovery of PTSs, including diffusible proteinaceous toxins associated with novel targeting modules (3, 13). One such system, dubbed the S8-associated polymorphic toxin system (S8-PTS), is encoded by a three-gene locus, comprising (i) a secreted S8 family serine peptidase (subtilase), (ii) a secreted variable toxin fused to a conserved N-terminal BetaH domain, and (iii) a cytosol-retained immunity protein. The S8-PTS is present in approximately 700 bacterial species across the Firmicutes and Actinobacteria (Actinomycetota) phyla, including human-associated organisms such as *Mycobacterium abscessus*, *Bacillus cereus*, *Staphylococcus aureus*, and *Listeria monocytogenes* (13).

Here, we investigate the S8-Ntox35 locus identified in *S. aureus* and provide experimental evidence that it encodes a bona fide diffusible proteinaceous toxin capable of mediating antagonism across genus boundaries—an activity that distinguishes it from previously described toxins and suggests a broader ecological role in competitive interactions within Firmicutes. We demonstrate that S8 peptidase processing reveals a caged amphipathic cationic element within the BetaH domain, which greatly enhances the activity of Ntox35. Finally, we identify a susceptibility determinant in the target organism required for intoxication and show that disruption of this transporter reveals an evolutionary tradeoff: resistance to Ntox35 is accompanied by increased susceptibility to antibiotics normally countered by the transporter, as well as heightened sensitivity to RNase-independent toxin activity.

## RESULTS

### The *S. aureus* S8-Ntox35 locus encodes an inter-genus toxin

**Fig 1:**
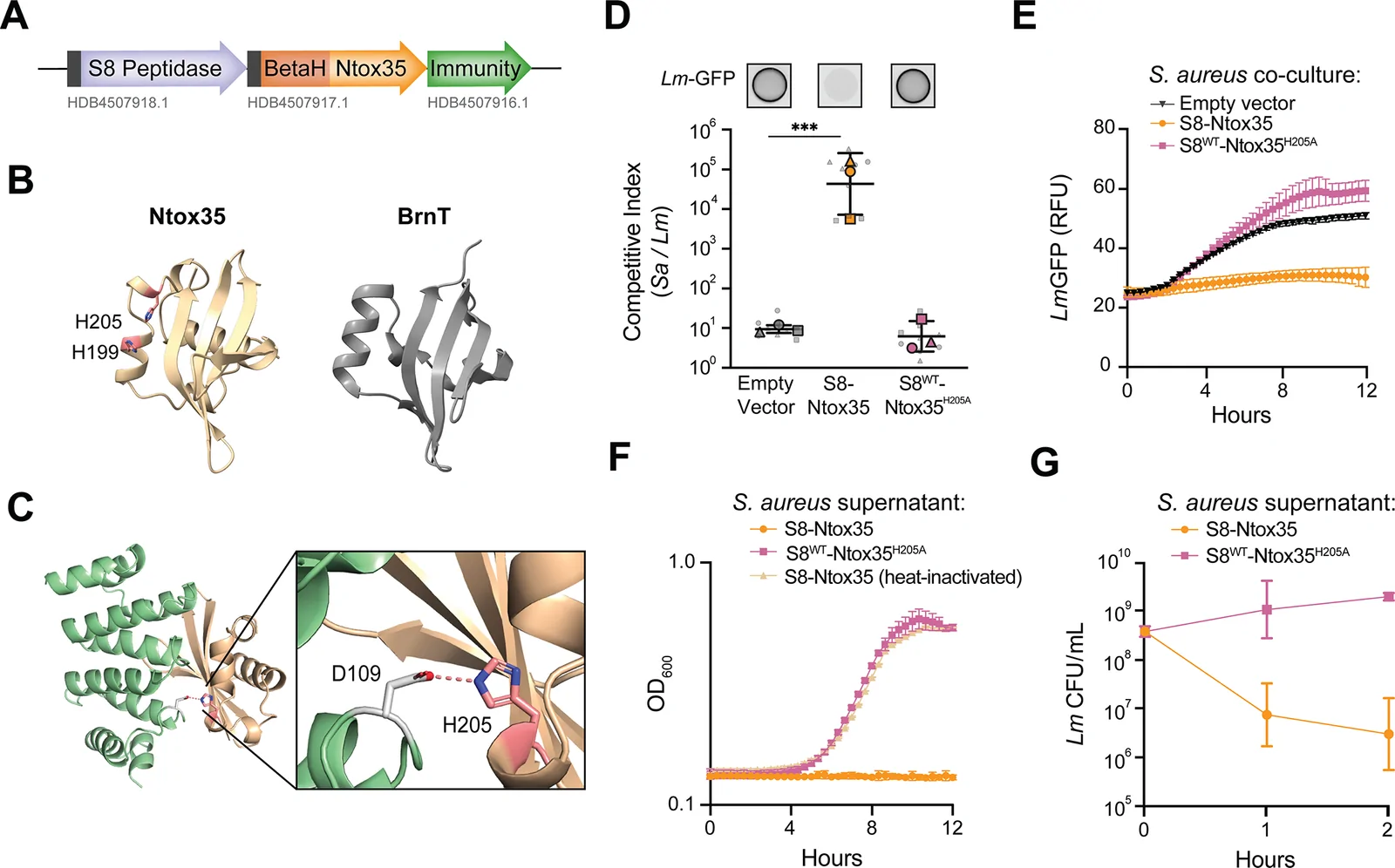
The S8-Ntox35 polymorphic toxin mediates diffusible interbacterial killing. (**A**) Organization of the S8-PTS locus from *S. aureus isolate* AMT0168-17, consisting of a secreted S8 peptidase (HDB4507918.1) containing a Sec signal sequence (black box), a Sec signal-containing BetaH domain fused to the variable toxin Ntox35 (HDB4507917.1), and the cognate immunity protein TPR-Imm1 (HDB4507916.1). (**B**) Predicted structure of the Ntox35 C-terminal toxin domain (amino acids 181–269), generated using AlphaFold 3 (pTM = 0.87), aligned to the structurally related RNase toxin BrnT (PDB: 3U97) identified using the Dali protein structure comparison server (Z = 6.7, RMSD = 2.7 Å, LALI = 70). Conserved catalytic histidine residues are highlighted. (**C**) AlphaFold 3 structural model of the interaction between the Ntox35 toxin domain (tan, right) and the cognate immunity protein TPR-Imm1 (green, left) (ipTM = 0.91, pTM = 0.58). Enlarged inset highlights the predicted interaction between Asp109 of TPR-Imm1 and His205 of Ntox35. Full-length BetaH-Ntox35 and BetaH-Ntox35–TPR-Imm1 models colored by pLDDT confidence are shown in Fig. S1A and B and provided as downloadable PDB files. (**D**) Contact-dependent competition between *S. aureus* strains encoding the indicated S8-Ntox35 loci and GFP-expressing *L. monocytogenes* (*Lm*-GFP) on solid agar. Competitive indices (CIs) were calculated from colony-forming units (CFU) recovered from mixed colonies and defined as (*S. aureus*_final_/*L. monocytogenes*_final_)/(*S. aureus*_initial_/*L. monocytogenes*_initial_). Representative images show GFP fluorescence from excised colonies. Data are presented as geometric mean ×/÷ geometric SD from three biological replicates (colored symbols, matched by shape), with technical replicates shown in gray. Asterisks indicate values significantly different from the empty-vector control (one-way ANOVA on log₁₀-transformed CI values with Dunnett’s multiple-comparison test). (**E**) Suspension competition between GFP-expressing *L. monocytogenes* (*Lm*-GFP) and *S. aureus* strains expressing the indicated S8-Ntox35 loci. GFP-associated relative fluorescence units (RFU) were measured every 20 min during coculture in a microplate reader. Data are presented as mean ± SD from three technical replicates and are representative of three independent biological experiments initiated from separate overnight cultures. (**F**) Growth of GFP-expressing *L. monocytogenes* (*Lm*-GFP), normalized to a starting density of approximately 5 × 10⁵ CFU mL^−^¹, following exposure to the indicated *S. aureus*-derived supernatants (2.5% [vol/vol]), including heat-inactivated wild-type supernatant (95°C for 10 min). Optical density at 600 nm (OD_600_) was measured every 20 min during incubation in a microplate reader. Data are presented as mean ± SD from three technical replicates and are representative of three independent biological experiments initiated from separate overnight cultures. (**G**) Survival of GFP-expressing *L. monocytogenes* (*Lm*-GFP) following treatment with supernatants derived from *S. aureus* expressing wild-type S8-Ntox35 or catalytic mutant Ntox35 (S8^WT^-Ntox35^H205A^) was determined by CFU enumeration over time. Data are presented as geometric mean ×/÷ geometric SD from three biological replicates. Asterisks indicate CFU values significantly different from the corresponding 0 h input for each treatment condition (one-way ANOVA on log_10_-transformed CFU values with Dunnett’s multiple-comparison test).

Because ~65% of genomes encoding the S8-PTS locus belong to the genus *Staphylococcus* (13), we selected *S. aureus* isolate AMT0168-17 (DALKLH000000000.1) as a model to investigate system function. Strains harboring this locus encode a secreted S8 family peptidase, a BetaH domain fused to a toxin domain termed Ntox35, and a cytosolic immunity protein, TPR-Imm1 (Fig. 1A).

Structural modeling of the Ntox35 C-terminal (pTM = 0.87), combined with similarity searches against the Protein Data Bank (PDB), identified homology to a characterized RNase toxin from *Brucella abortus* (Dali Z = 6.7; PDB: 3U97) (Table S5). This is consistent with prior analyses predicting that Ntox35 adopts a canonical BECR (Barnase–EndoU–Colicin E5/D–RelE) ribonuclease fold composed of an N-terminal α-helix followed by a four-stranded antiparallel β-sheet (αββββ) (Fig. 1B) (3, 14). Conserved histidine residues within this helix often participate in catalysis (3, 13), corresponding to H199 and H205 in Ntox35 (Fig. 1B). AlphaFold 3 modeling of the Ntox35–TPR-Imm1 complex (ipTM = 0.91, pTM = 0.58) predicts interaction between Asp109 of the immunity protein and His205 of Ntox35 (Fig. 1C), consistent with active site occlusion (15). Together, these features support the hypothesis that Ntox35 functions as an RNase toxin.

Despite this bioinformatic evidence, Ntox35 had not been experimentally validated as an antibacterial effector. In an intra-species competition assay, *S. aureus* expressing the full S8-Ntox35 locus did not inhibit the growth of an isogenic GFP-labeled *S. aureus* strain lacking the locus (Fig. S1C). In contrast, co-culture with *L. monocytogenes* 10403S constitutively expressing GFP (*Lm*-GFP) resulted in robust growth inhibition (Fig. S1C) and an approximately 4-log increase in competitive fitness of the toxin-producing strain (Fig. 1D). Mutation of a predicted catalytic residue (H205A) abolished this activity, demonstrating that antagonism requires Ntox35 catalytic function (Fig. 1D). These findings indicate that the S8-Ntox35 locus mediates inter-genus competition, in contrast to many previously described PTSs.

Both the S8 peptidase and the BetaH-Ntox35 fusion contain N-terminal signal peptides consistent with Sec-dependent secretion, whereas the immunity protein lacks a signal peptide, suggesting cytosolic retention (3, 13, 16, 17). The toxin could therefore function as a surface-associated effector or as a diffusible inhibitor. To distinguish between these possibilities, we performed competition assays under shaking liquid culture conditions that limit stable cell-cell contact. Under these conditions, *S. aureus* expressing wild-type S8-Ntox35 continued to inhibit the growth of *L. monocytogenes*, whereas the H205A mutant did not (Fig. 1E), consistent with diffusible activity of the Ntox35 toxin domain.

To directly test whether inhibition was mediated by a secreted factor, we collected and concentrated cell-free supernatants from toxin-producing strains. Supernatants containing wild-type S8-Ntox35 retained potent inhibitory activity against *L. monocytogenes*, whereas those from the H205A mutant did not. Heat treatment at 95°C abolished this activity, indicating that the observed inhibition of *L. monocytogenes* growth is mediated by a heat-labile protein (Fig. 1F; Fig. S1D). Exposure of *L. monocytogenes* to active S8-Ntox35 supernatant resulted in a ~100-fold reduction in viable counts within 2 h relative to the H205A control (Fig. 1G), demonstrating a bactericidal effect. Consistent with the predicted RNase activity of Ntox35, total RNA extracted from intoxicated cells showed accumulation of low-molecular-weight fragments, which were absent following treatment with catalytically inactive supernatant (Fig. S1E). Together, these data establish that the S8-Ntox35 locus encodes a secreted, diffusible RNase toxin capable of mediating inter-genus antagonism.

### Toxin activation requires S8 peptidase processing

**Fig 2:**
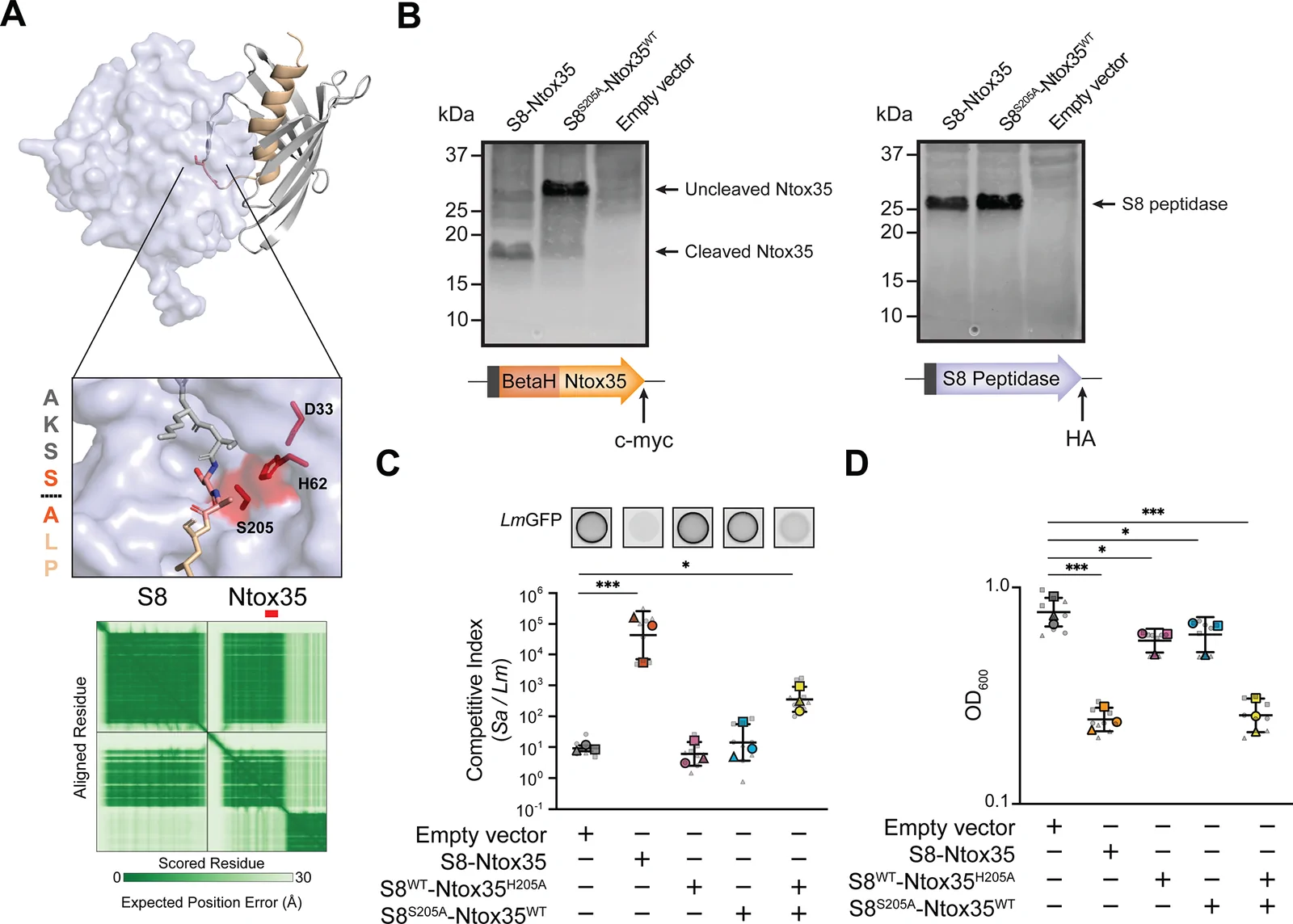
S8-peptidase proteolytic cleavage enhances Ntox35 activity. (**A**) AlphaFold 3 multimer modeling of the S8 peptidase (blue surface representation) in complex with the cognate BetaH domain (β-sheet in gray, α-helix in tan ribbon representation) predicted a high-confidence interaction (ipTM = 0.79, pTM = 0.69). The predicted aligned error (PAE) plot is shown below, with the predicted interaction interface between the S8 peptidase and BetaH domain highlighted by the red box. The S8 peptidase active site is positioned adjacent to a flexible loop connecting the β-sheet and α-helical regions of the BetaH domain. Enlarged inset shows alignment of the S8 catalytic triad (D33, H62, and S205) in a configuration consistent with proteolytic cleavage between S146 and A147 of the BetaH domain. Putative cleavage site residues are indicated, with the predicted cleavage site marked by a dashed line. (**B**) Western blot analysis of concentrated supernatants from *S. aureus* strains expressing wild-type S8-Ntox35 (S8^WT^-Ntox35^WT^), the peptidase mutant (S8^S205A^-Ntox35^WT^), or empty vector. C-terminal Myc and HA epitope tags were fused to Ntox35 and the S8 peptidase, respectively. Immunoblotting revealed a lower-molecular-weight Ntox35 cleavage product in the wild-type supernatant, whereas only full-length toxin was detected in the peptidase mutant supernatant, indicating that processing requires S8 peptidase catalytic activity. (**C**) Contact-dependent competition between *S. aureus* strains encoding the indicated S8-Ntox35 loci and GFP-expressing *L. monocytogenes* (*Lm*-GFP) on solid agar. Competitive indices (CIs) were calculated from CFUs recovered from mixed colonies and defined as (*S. aureus*_final_/*L. monocytogenes*_final_)/(*S. aureus*_initial_/*L. monocytogenes*_initial_). Representative images show GFP fluorescence from excised colonies. Data are presented as geometric mean ×/÷ geometric SD from three biological replicates (colored symbols, matched by shape), with technical replicates shown in gray. Asterisks indicate values significantly different from the empty-vector control (one-way ANOVA on log_10_-transformed CI values with Dunnett’s multiple-comparison test). (**D**) Growth of GFP-expressing *L. monocytogenes* (*Lm*-GFP), normalized to a starting OD_600_ of 0.25, following treatment with the indicated *S. aureus*-derived supernatants (2.5% vol/vol). Conditions included wild-type S8-Ntox35, catalytic mutant Ntox35, peptidase mutant Ntox35, and a 1:1 mixture of catalytic and peptidase mutant supernatants. OD_600_ was recorded every 20 min during incubation, and values at 4 h—corresponding to stationary phase in untreated cultures—were used for comparisons. Data are presented as mean ± SD from three biological replicates (colored symbols, matched by shape), with technical replicates shown in gray. Asterisks indicate values significantly different from treatment with empty-vector control supernatant (one-way ANOVA with Dunnett’s multiple-comparison test).

S8 peptidases (subtilases) are broadly conserved serine proteases that often mediate proteolytic processing of associated substrates. In bacteria, these enzymes typically function as proprotein-processing endopeptidases or are fused to components of established secretion pathways. In contrast, S8 peptidases associated with BetaH-containing polymorphic toxins lack a prodomain and possess an N-terminal Sec signal peptide, suggesting extracellular localization of a mature enzyme (13, 18, 19). Structural modeling of the S8 peptidase–BetaH-Ntox35 complex (ipTM = 0.79, pTM = 0.69) predicts that a flexible loop separating the β-sheet and α-helix elements of the BetaH domain is positioned within the peptidase active site (Fig. 2A). In this model, the peptidase’s catalytic serine (S205) is oriented to mediate hydrolysis between residues S146 and A147, consistent with cleavage within the BetaH domain.

To determine whether S8 mediates processing of the BetaH-Ntox35 fusion, we introduced a catalytic serine-to-alanine substitution (S8^S205A^-Ntox35^WT^) while maintaining a wild-type toxin sequence. HA and Myc epitope tags were appended to the C-termini of the S8 peptidase and Ntox35, respectively, to distinguish cleaved and full-length products. Supernatants from the wild-type locus contained an ~18 kDa Ntox35 species, whereas those from the S8^S205A^ mutant contained a ~30 kDa product corresponding to the uncleaved BetaH-Ntox35 fusion (Fig. 2B). These data indicate that the S8 peptidase mediates proteolytic cleavage of the toxin precursor.

We next assessed whether S8-dependent processing plays a role in target cell intoxication. *S. aureus* expressing wild-type Ntox35 but harboring the catalytic S8^S205A^ mutation failed to inhibit growth of susceptible *L. monocytogenes* in both co-culture assays and upon treatment with concentrated cell-free supernatants (Fig. 2C and D; Fig. S2). To test whether processing can occur in *trans*, we co-cultured strains expressing inactive toxin (S8^WT^-Ntox35^H205A^) with strains expressing inactive peptidase (S8^S205A^-Ntox35^WT^) and similarly mixed their concentrated supernatants. In both cases, growth inhibition was restored (Fig. 2C and D; Fig. S2), indicating extracellular complementation between active S8 peptidase and wild-type toxin. Together, these results demonstrate that S8-dependent proteolytic processing occurs extracellularly and is important for Ntox35 activity.

### Bacterial surface charge resists S8-PTS intoxication

**Fig 3:**
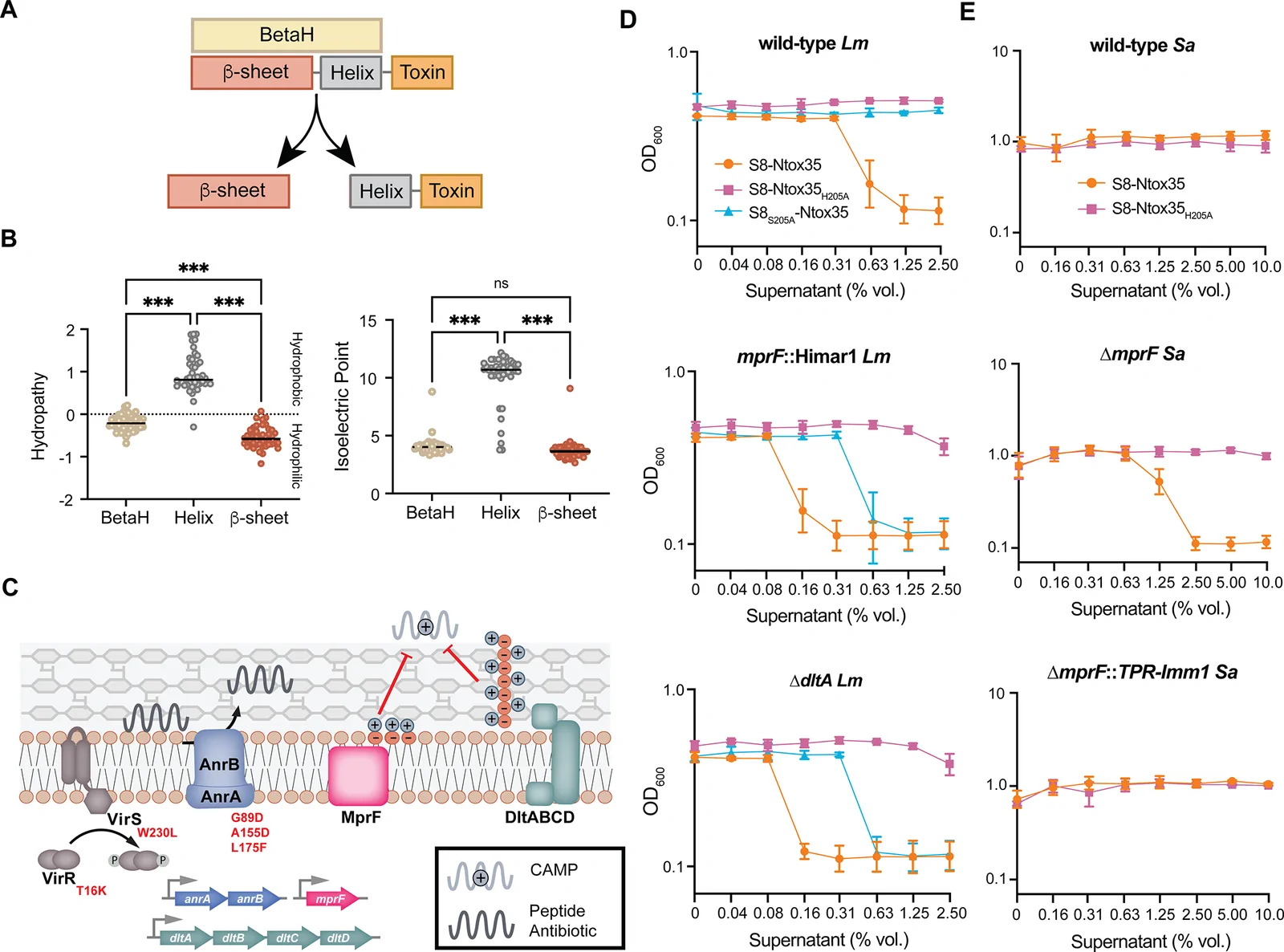
Surface charge modulates toxin susceptibility. (**A**) Schematic of the predicted S8-mediated cleavage event liberating the N-terminal β-sheet domain (red) from the C-terminal α-helical tail (gray) and fused Ntox35 effector domain (orange). (**B**) Grand average hydropathy and predicted isoelectric point values for 44 intact BetaH domains (tan), the S8-liberated N-terminal β-sheet fragment (red), and the C-terminal α-helical fragment released with the toxin effector (gray). (**C**) Model of the VirRS-AnrAB cell envelope stress response pathway. Activation of the VirRS two-component system induces expression of the ABC transporter AnrAB and downstream cell envelope modification pathways, including *mprF* and *dltABCD. mprF* mediates lysinylation of phosphatidylglycerol, while *dltABCD* promotes D-alanylation of teichoic acids, collectively reducing the net negative surface charge of the bacterial envelope and limiting interaction with antimicrobial peptides. Mutations identified in experimentally evolved S8-Ntox35-resistant isolates are indicated in red. (**D and E**) Growth of indicated (**D**) *L. monocytogenes* or (**E**) *S. aureus* strains, normalized to a starting density of approximately 5 × 10⁵ CFU mL^−^¹ and treated with titrated amounts of the indicated *S. aureus*-derived supernatants. OD_600_ was recorded every 20 min during incubation, and values at 10 h (*L. monocytogenes*) or 6 h (*S. aureus*)—corresponding to stationary phase in untreated cultures—were used for comparisons. Data are presented as mean ± SD from three biological replicates. Asterisks indicate values significantly different from the respective wild-type strain at the indicated supernatant concentration (two-way ANOVA with Dunnett’s multiple-comparison test).

*In silico* structural modeling of the S8 peptidase–Ntox35 complex predicts that the BetaH domain adopts a bipartite architecture in which a strongly anionic β-sheet interfaces with an α-helix enriched in basic residues. A flexible loop connecting these elements is positioned adjacent to the Asp/Ser/His catalytic triad of the S8 peptidase active site (Fig. 2A). A similar spatial arrangement is predicted for other S8-PTS toxin-peptidase pairs (Fig. S3), suggesting a conserved mode of interaction. Together with our biochemical evidence of toxin cleavage, these data support a model in which S8-mediated proteolysis separates the N-terminal β-sheet from the α-helix and the downstream Ntox35 domain (Fig. 3A), generating a processed toxin with an exposed N-terminal helical extension.

Because this α-helical region is enriched in basic and hydrophobic residues, we assessed how its physicochemical properties differ from those of the intact BetaH domain. Across 44 representative BetaH-toxin fusion proteins, the predicted hydrophobicity and isoelectric point (pI) of the helix were consistently higher than those of the intact BetaH domain or isolated β-sheet (Fig. 3B). These features are consistent with properties of cationic antimicrobial peptides (CAMPs), suggesting that processed Ntox35 may engage bacterial membranes through electrostatic interactions.

In Firmicutes, resistance to CAMPs is commonly mediated by surface charge modification, which generates a cationic barrier that repels positively charged molecules. Two well-characterized systems mediate this effect: MprF, which lysinylates phosphatidylglycerol, and DltABCD, which mediates D-alanylation of teichoic acids (Fig. 3C) (20–22). To test whether these pathways influence susceptibility to Ntox35, we challenged wild-type *L. monocytogenes*, a Δ*dltA* mutant (23), and an *mprF* transposon mutant (24) with titrated amounts of S8-Ntox35-containing supernatant. Both mutants exhibited increased sensitivity relative to wild type, with growth inhibition occurring at 0.16% (vol/vol) supernatant compared to 0.63% for wild type (Fig. 3D; Fig. S4A).

We next tested supernatant containing unprocessed Ntox35 (S8^S205A^-Ntox35^WT^) and observed that both mutants were inhibited at 0.63% (vol/vol) (Fig. 3D; Fig. S4B), a concentration that did not affect the wild-type strain. This indicates that reduced surface charge enhances susceptibility even in the absence of S8-mediated processing. Exposure to processed but catalytically inactive toxin (S8^WT^-Ntox35^H205A^) resulted in modest growth inhibition at 2.5% (vol/vol) (Fig. 3D; Fig. S4C), indicating that processed toxin retains low-level activity at the cell surface independent of RNase function.

These results prompted us to re-examine our earlier observation that *S. aureus* (lacking the S8-PTS locus) appeared resistant to intoxication (Fig. S1C). When tested in the same supernatant titration assay used in Fig. 3D, wild-type *S. aureus* displayed markedly greater resistance than wild-type *L. monocytogenes*; whereas *L. monocytogenes* growth was inhibited at 0.63% supernatant, *S. aureus* exhibited no detectable growth defect even at 10%. However, deletion of *mprF* in *S. aureus* significantly sensitized the strain, with growth inhibition observed at 2.5%. Introduction of TPR-Imm1 into the *mprF* deletion strain restored growth to wild-type levels (Fig. 3E; Fig. S5A and B), confirming that the observed sensitization reflects toxin-specific activity rather than generalized envelope stress.

### Intoxication by S8-Ntox35 requires the ABC transporter AnrAB

**Fig 4:**
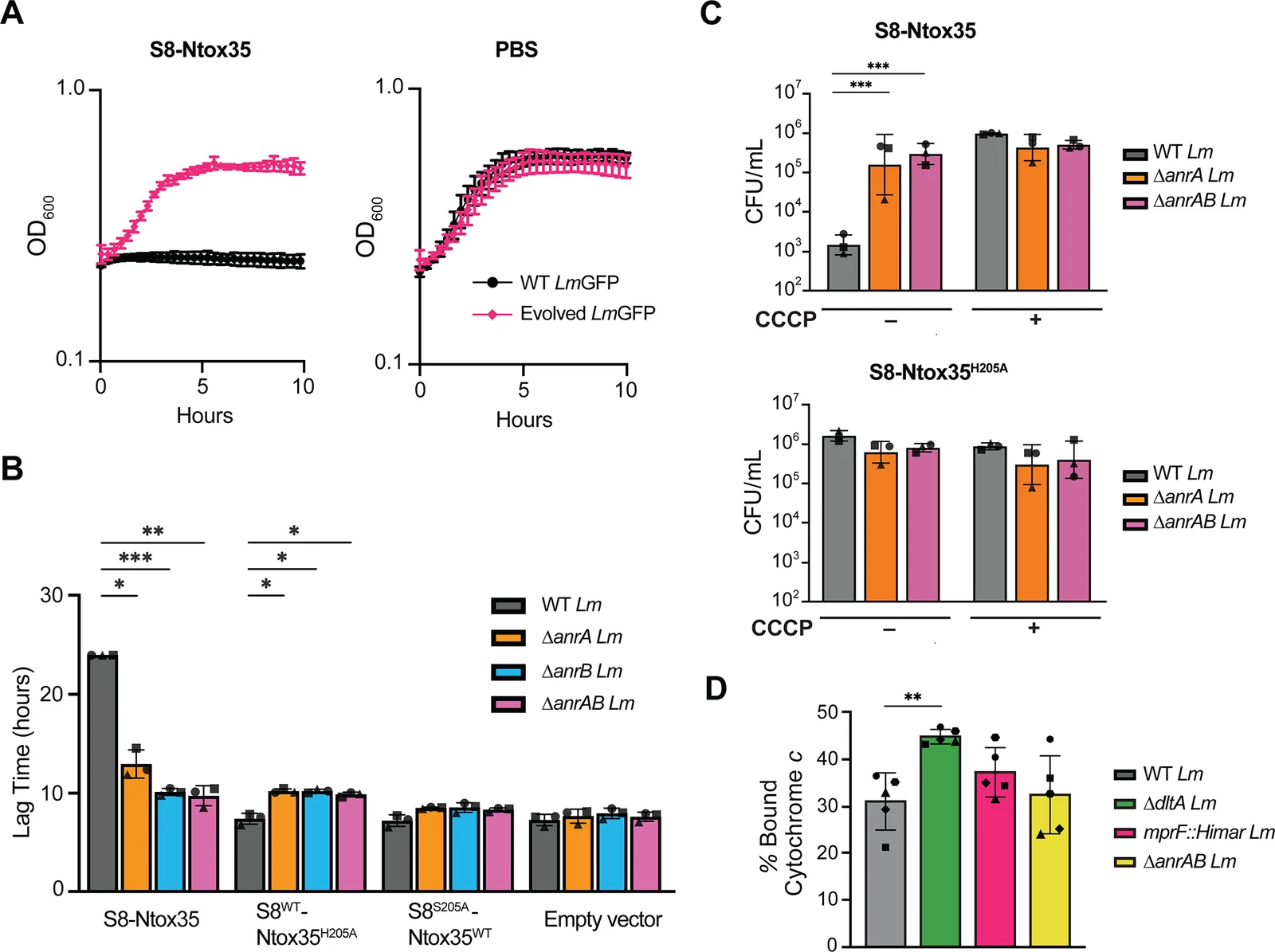
The AnrAB transporter is required for S8-Ntox35 intoxication. (**A**) Growth of sensitive GFP-expressing *L. monocytogenes* (*Lm*-GFP) and experimentally evolved *Lm*-GFP following exposure to concentrated supernatant from *S. aureus* expressing wild-type S8-Ntox35 (left) or PBS vehicle control (right). Data are presented as mean ± SD from three technical replicates. (**B**) Lag time estimates for the indicated *L. monocytogenes* strains following treatment with *S. aureus*-derived supernatant. Growth curves were fitted using a modified Baranyi–Roberts model, and bars represent mean lag time ± SD derived from fitted parameters. Asterisks indicate lag time (within treatment) significantly different from wild-type *L. monocytogenes* (one-way ANOVA with Dunnett’s multiple-comparison test). (**C**) Survival of the indicated *L. monocytogenes* strains following 2 h exposure to *S. aureus*-derived supernatant in the presence or absence of the membrane-depolarizing agent CCCP. Viability was determined by CFU enumeration. Asterisks indicate values significantly different from wild-type *L. monocytogenes* (two-way ANOVA with Dunnett’s multiple-comparison test). (**D**) Relative cytochrome *c* binding by wild-type *L. monocytogenes* and the Δ*dltA*, *mprF::Himar1*, and Δ*anrAB* mutants. Percent cytochrome *c* binding was calculated relative to cytochrome *c*-only controls. Data are presented as mean ± SD from five biological replicates. Asterisks indicate values significantly different from wild-type *L. monocytogenes* (one-way ANOVA with Dunnett’s multiple-comparison test).

To identify additional determinants of susceptibility to Ntox35, we subjected sensitive wild-type *L. monocytogenes* (*Lm*-GFP) to experimental evolution under subinhibitory concentrations of active Ntox35-containing supernatant. After three passages, a resistant population emerged (Fig. 4A). Whole-genome sequencing of nine independent clones revealed that all carried missense mutations in components of the VirRS-AnrAB module, including mutations in *virS* (*n* = 1), *virR* (*n* = 1), and *anrA* (*n* = 7) (Fig. 3C; Table S1). Additional mutations were only observed in combination with *anrA* variants, suggesting strong selective pressure on AnrAB function.

VirRS regulates genes involved in cell envelope modification and antimicrobial peptide resistance, including *mprF*, *dltABCD*, and the ATP-binding cassette (ABC) transporter *anrAB* (25–27). Given the CAMP-like features of Ntox35, we hypothesized that disruption of AnrAB would increase susceptibility to intoxication (27–32). To test this, we generated in-frame deletions of *anrA*, *anrB*, and both genes in *L. monocytogenes*. As expected, all mutants exhibited reduced resistance to the antimicrobial peptide bacitracin, with the MIC decreasing from 63.5 µg/mL in wild-type to 8 µg/mL in transporter mutants (28–30). Unexpectedly, however, loss of AnrAB reduced sensitivity to Ntox35: lag time following intoxication decreased from >20 h in wild-type to ~13 h in Δ*anrA* and ~10 h in Δ*anrB* and Δ*anrAB* strains (Fig. 4; Fig. S6). These findings indicate that AnrAB is required for full susceptibility to Ntox35, consistent with a role in toxin entry rather than detoxification. Disruption of the ATPase component (AnrA) alone was sufficient to confer protection, suggesting that transporter activity is required (33). Although typically associated with detoxification, ABC transporters can mediate either efflux or import depending on substrate and context and have been implicated in translocation of certain CDI toxins across the inner membrane of *Escherichia coli* (33, 34).

Prior work demonstrated that proton motive force (PMF) is required for cytoplasmic entry of S8-PTS toxins (35). Because PMF is necessary for ATP synthesis in bacteria, treatment with the uncoupler carbonyl cyanide-*m*-chlorophenylhydrazone (CCCP) reduces cellular ATP levels and can impair ATP-dependent processes, such as ABC transporter activity. We therefore tested whether PMF dependence overlaps with the requirement for AnrAB. Wild-type, Δ*anrA*, and Δ*anrAB* strains were intoxicated in the presence or absence of CCCP. Consistent with previous work by Colautti et al., CCCP significantly increased resistance of wild-type cells (35); however, we observed no additional effect in Δ*anrA* or Δ*anrAB* strains (Fig. 4C; Fig. S7). Although CCCP has pleiotropic effects, this non-additive interaction suggests that PMF-dependent ATP production and AnrAB function operate within the same intoxication pathway, consistent with ATP-dependent AnrAB activity contributing to toxin susceptibility.

Although Δ*anr* mutants were protected from the pronounced growth delay observed in wild-type cells, protection was incomplete. Baseline lag times in control treatments (empty vector and PBS) were tightly distributed between ~7.0 and 8.0 h. Exposure to uncleaved, catalytically active toxin produced a modest but consistent increase in lag (~8.4–8.6 h), representing a small shift relative to baseline. In contrast, processed toxin, both catalytically active and inactive, resulted in substantially longer lag times (~9.9–10.2 h and ~9.8–13.0 h, respectively; Fig. 4B; Fig. S6). The similar lag observed for processed toxin regardless of catalytic activity indicates that RNase function does not account for most of this residual delay. Together, these data suggest that AnrAB-dependent cytosolic intoxication drives the dominant phenotype, whereas processed toxin imposes a secondary, RNase-independent growth cost consistent with membrane-associated effects.

**Fig 5:**
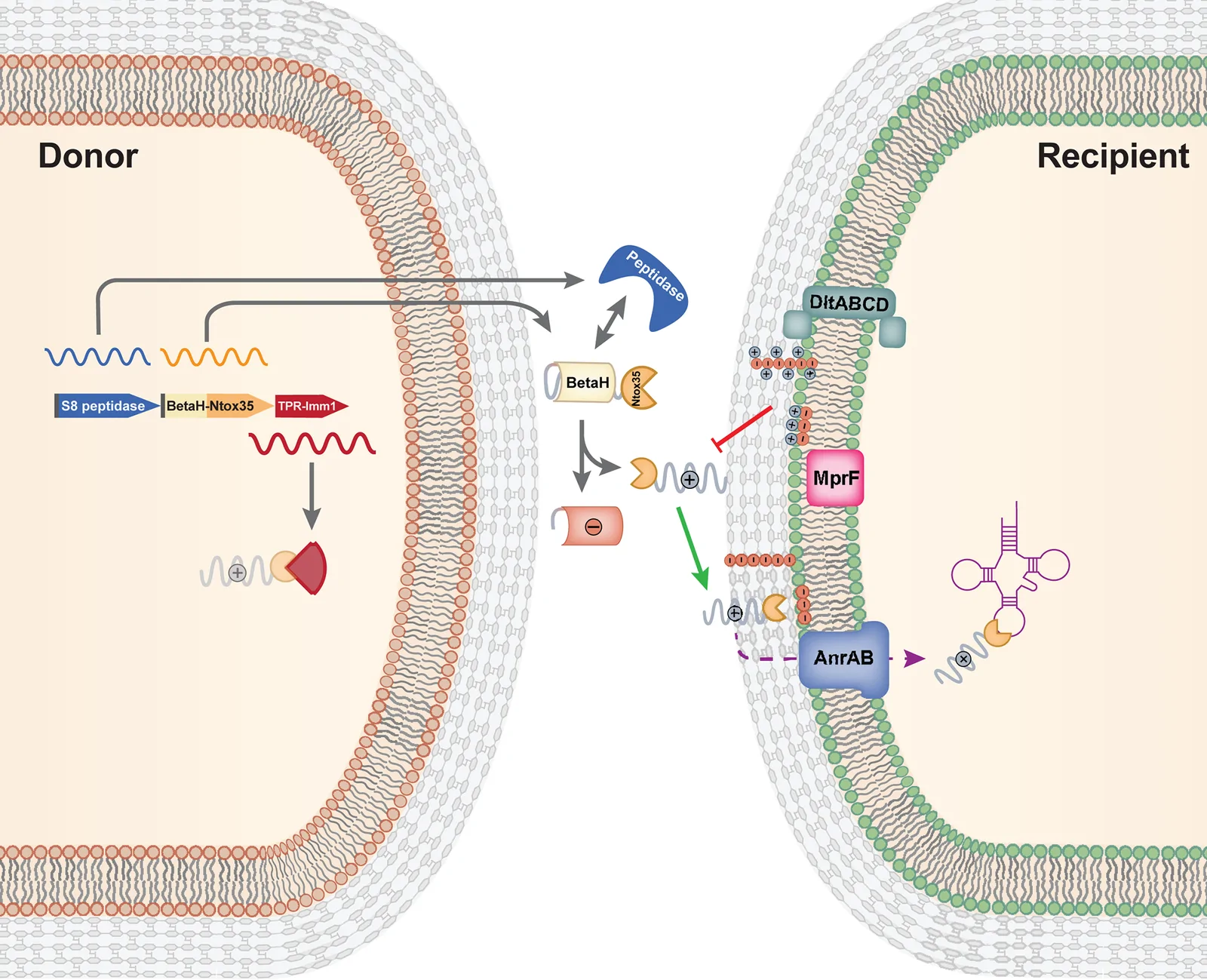
Proposed model for S8-Ntox35 processing, resistance, and intoxication. The S8-Ntox35 locus encodes an S8 peptidase, a BetaH-Ntox35 toxin precursor, and the cognate immunity protein TPR-Imm1. In the producing organism, TPR-Imm1 binds Ntox35 to provide self-protection. The S8 peptidase and BetaH-Ntox35 precursor are secreted extracellularly, where the peptidase cleaves the BetaH domain to release a processed toxin containing a positively charged α-helical tail fused to the Ntox35 effector domain. In recipient cells, membrane modifications mediated by *mprF* and *dltABCD* reduce surface negative charge and are proposed to repel the processed toxin. In susceptible cells, the toxin is attracted to negatively charged membranes, where AnrAB is proposed to facilitate toxin translocation across the membrane (dashed arrow). Once internalized, Ntox35 is proposed to target cellular RNA.

The residual RNase-independent toxicity observed in Δ*anr* strains resembled the phenotype previously observed in Δ*dltA* and *mprF* mutants. To distinguish whether this similarly reflected surface charge interactions, we compared cytochrome *c* binding across these strains. As expected, Δ*dltA* cells exhibited significantly increased binding relative to wild-type, consistent with a more negatively charged surface. The *mprF* mutant displayed a modest but non-significant increase, whereas Δ*anrAB* cells were comparable to wild type (Fig. 4D). These results indicate that the phenotype of Δ*dltA* (and to a lesser extent the *mprF* mutant) strains likely reflects increased electrostatic recruitment of toxin to the cell surface. In contrast, because Δ*anrAB* does not exhibit appreciable changes in surface charge yet retains a residual growth delay upon toxin exposure, its phenotype is more consistent with impaired translocation and accumulation of toxin at the membrane rather than increased surface binding (Fig. 5).

## DISCUSSION

In this study, we define the molecular mechanism of S8-PTS-mediated intoxication by the RNase toxin Ntox35 in *S. aureus*. We show that this system mediates inter-genus antagonism via a diffusible, heat-labile protein whose bactericidal activity depends on a catalytic residue within Ntox35. We further demonstrate that processing by the associated S8 peptidase greatly enhances toxicity, that target cell surface charge modulates susceptibility, and that the ABC transporter AnrAB is required for efficient intoxication in *L. monocytogenes*. Because the S8 peptidase and BetaH domain are conserved across S8-PTS loci, these mechanistic features are likely shared among related systems.

Experimental characterization of diffusible polymorphic toxins has largely focused on colicins in *E. coli* and soluble pyocins in *Pseudomonas*, which typically exhibit narrow target spectra (3, 7, 11, 12). In contrast, diffusible antibacterial effectors in gram-positive bacteria have largely been limited to small peptides rather than multi-domain polymorphic toxins (3, 12, 36). The S8-PTS therefore represents an unusual architecture, combining a modular toxin framework with diffusible activity and broad target range. While our work demonstrates inter-genus antagonism, recent findings suggest that S8-PTS activity may extend across phyla (35).

We demonstrate that toxin activity is greatly enhanced following extracellular proteolytic processing by a separately encoded S8 peptidase. Complementary work by Colautti et al. independently mapped the cleavage site, supporting this model (35). This arrangement contrasts with previously described polymorphic toxins in which processing domains are embedded within the toxin itself (3, 13). The conserved pairing of the S8 peptidase and the BetaH domain across S8-PTS loci indicates that this processing step is a mechanistically central feature of this system.

The BetaH domain has not previously been associated with polymorphic toxins; however, in the S8-PTS loci, it is fused to diverse C-terminal toxin domains, including RNases and DNases (10). Processing of the BetaH-Ntox35 precursor is predicted to expose a cationic, amphipathic N-terminal extension. Consistent with this, disruption of *dltA* and *mprF* increased susceptibility in both *L. monocytogenes* and *S. aureus*, indicating that surface charge modulates intoxication efficiency. These results support the hypothesis that S8-mediated cleavage exposes a membrane-targeting module analogous to CAMPs. This charge-dependent modulation may provide an immunity-independent resistance mechanism and may help explain the variable susceptibility across taxa noted by Colautti et al. (35).

A particularly unexpected finding was that disruption of the ABC transporter AnrAB reduced susceptibility to Ntox35. Although AnrAB is associated with resistance to cell envelope-active antimicrobials (26–30), Δ*anrAB* mutants displayed reduced growth inhibition, indicating that transporter activity contributes to intoxication. While ABC transporters have been implicated in toxin entry in other systems, ATPase activity is often dispensable (33, 34, 37). In contrast, our data indicate that AnrA-mediated ATP hydrolysis is required for efficient S8-Ntox35 intoxication. Together with the observation that CCCP protects wild-type but not Δ*anr* strains, these results suggest that ATP-dependent AnrAB activity facilitates cytoplasmic delivery of the RNase domain following membrane engagement.

Our data further indicate that S8-Ntox35 intoxication involves at least two mechanistically distinct layers. Cytosolic RNA degradation accounts for the bactericidal phenotype, whereas S8-processed toxin retains residual growth-inhibitory activity that is enhanced in *dltA* and *mprF* mutants and persists in Δ*anrAB* backgrounds. This indicates that membrane-targeting and RNase-dependent cytotoxicity are separable functions. Consistent with prior truncation analyses by Colautti et al. (35), these findings support a model in which the processed N-terminal extension mediates membrane engagement and contributes to RNase-independent growth inhibition, while AnrAB-dependent translocation enables full cytosolic toxicity.

The phylogenetic distribution of S8-PTS loci overlaps with that of Bce-like transporter modules in Firmicutes, raising the possibility that conserved envelope surveillance systems are exploited for toxin entry (31, 32, 38). However, sequence divergence and susceptibility of organisms lacking clear AnrAB homologs suggest that transporter dependence may vary across taxa (31, 39). One possibility is that S8-PTS toxins engage a conserved membrane ligand, such as a lipid intermediate involved in cell wall biosynthesis that is protected by Bce modules, and that the ability of these modules to disrupt antimicrobial binding to these lipid targets may facilitate translocation in certain hosts. Combined with our findings establishing envelope charge repulsion as a susceptibility determinant, this model provides a framework for understanding how a diffusible RNase toxin achieves a broad but non-universal target range.

Taken together, our findings support a multi-step model of S8-Ntox35 intoxication: extracellular proteolytic activation, charge-dependent membrane engagement, and a proposed role for AnrAB-facilitated delivery of the RNase domain into the cytosol of *L. monocytogenes* (Fig. 5). By integrating proteolytic activation, CAMP-like targeting, and transporter-dependent entry, the S8-PTS expands the mechanistic diversity of polymorphic toxin systems in gram-positive bacteria. Given the conservation of the S8 peptidase-BetaH architecture, these principles are likely broadly applicable. Future work will define the molecular basis of transporter engagement, identify membrane ligands that promote toxin recruitment, and determine how broadly transporter dependence extends across species.

## MATERIALS AND METHODS

### Use of artificial intelligence tools

Portions of the code used for growth curve analysis were developed with assistance from ChatGPT (OpenAI) as an interactive programming aid. All analytical approaches, parameter selection, and statistical methods were designed, validated, and interpreted by the authors, and all final code was reviewed and executed by the authors.

ChatGPT (OpenAI) was used to assist with language editing, improve clarity and readability, and refine the organization of the manuscript during revision. All scientific content, experimental design, data analysis, interpretation of results, and final editorial decisions were performed and verified by the authors, who take full responsibility for the content of the manuscript.

### Quantification and statistical analysis

Statistical significance was defined as *P* < 0.05. Exact tests, replicate numbers, and definitions of center and error bars are provided in the figure legends.

### Bacterial strains and culture conditions

All bacterial strains used in this study are summarized in Table S2. As indicated, bacteria were cultured on brain heart infusion (BHI) (RPI B11000-5000.0) agar (*L. monocytogenes* and *S. aureus*), tryptic soy agar (TSA) (BD), or LB (Miller) agar (Fisher Scientific BP97235) (*L. monocytogenes*, *S. aureus*, and *E. coli*) at 37°C. Unless otherwise stated, prior to experiments, bacteria were grown in BHI broth (*L. monocytogenes* and *S. aureus*) or LB broth (*E. coli*) overnight at 37°C with shaking (220 rpm). Antibiotics (RPI) were used at the following concentrations: streptomycin, 200 μg/mL (*L. monocytogenes*); chloramphenicol, 10 μg/mL (*E. coli* and *S. aureus*); erythromycin, 2 µg/mL (*L. monocytogenes*); and carbenicillin, 100 μg/mL (*E. coli*). Plasmids were introduced into *E. coli* by chemical transformation, into *L. monocytogenes* by trans-conjugation, and into *S. aureus* by electroporation.

### Plasmid and strain construction

Plasmids and oligonucleotides used in this study are listed in Tables S3 and S4, respectively. Primers and synthetic DNA fragments were obtained from Integrated DNA Technologies (IDT). All plasmid constructs were designed using SnapGene and generated using Gibson Assembly, In-Fusion cloning, or restriction enzyme cloning, and all constructs were confirmed by sequencing. Amino acid substitutions were generated using overlapping site-directed mutagenesis primers. Substitutions were confirmed by PCR, Sanger sequencing, and whole plasmid sequencing. In-frame unmarked gene deletions in *L. monocytogenes* were generated via allelic exchange using plasmid pLIM followed by counterselection on 18 mM DL-4-chlorophenylalanine (40) and were confirmed by Sanger sequencing of PCR amplicons spanning the gene disruption. Plasmids were introduced to *L. monocytogenes* via trans-conjugation from *E. coli* SM10 (41). In-frame unmarked gene deletions in *S. aureus* were generated via allelic exchange using plasmid pIMAY*-Z (42) and inserts were confirmed by PCR and Sanger sequencing. Plasmid DNA was extracted from *E. coli* IM08B and introduced to *S. aureus* by electroporation and maintained by antibiotic selection (43–45). pEPSA5 (46) was used to express S8-Ntox35 locus constructs in *S. aureus*, with xylose induction of the plasmid.

### *In silico* analysis of S8-PTS components

Structural predictions of individual proteins and protein-protein complexes were conducted using AlphaFold 3 (47); the best-ranked model is displayed, and the corresponding PDB is provided in the supplemental materials. To identify structural similarities, amino acids 181–269 of Ntox35 were subjected to pairwise comparisons against a representative subset of the Protein Data Bank (PDB) using the default settings on the Dali server’s PDB25 search (http://ekhidna2.biocenter.helsinki.fi/dali/) (48). The top 10 hits are listed in Table S5. Protein sequences used for analysis of interactions between BetaH-toxins and cognate immunity and S8 peptidases are provided in Table S6. To determine the hydropathy and isoelectric points of intact BetaH domains and the two fragments generated by S8 protease processing, the sequences of 44 distinct BetaH toxins described by Li et al. were used (10). Analyzed sequences are provided in Table S7. Isoelectric point was calculated using IPC (49), and grand average of hydropathy was determined using the GRAVY calculator (www.gravy-calculator.de).

### Preparation of concentrated supernatant for S8-Ntox35 toxicity assays

*S. aureus* strains harboring either pEPSA5::S8-Ntox35-TPR_Imm1 or a corresponding mutant construct were used to produce toxin- and peptidase-containing supernatant (46). A single colony was inoculated into 100 mL of BHI broth and incubated, without xylose induction of pEPSA5, at 37°C with shaking for 20 h. Cultures were centrifuged at 4,500 × *g* for 20 min to pellet the cells. The resulting supernatant was sterile-filtered through a 0.2 µm vacuum-driven membrane filter (Thermo Scientific 565-0020) and then concentrated using a 10 kDa molecular weight cutoff Amicon Ultra Centrifugal Filter Unit (Millipore UFC901024) until the volume was reduced to approximately 2.5 mL. The concentrate was buffer exchanged into PBS using a Cytiva PD-10 column (17-0851-01) following the manufacturer’s instructions and sterile-filtered through a 0.2 µm syringe filter (Sterlitech 1470353). The final product was aliquoted, snap-frozen in liquid nitrogen, and stored at −80°C until further use.

### Measurement of S8-Ntox35 susceptibility

Stationary-phase overnight cultures were normalized to equivalent starting cell densities using one of two approaches, as indicated: cultures were adjusted either to an optical density at 600 nm (OD₆₀₀) of 0.25 or diluted to approximately 5 × 10⁵ CFU mL^−^¹ in fresh BHI or the relevant growth medium. For each condition, 80 µL of the normalized culture was dispensed into wells of a sterile 96-well cell culture plate (GenClone 25-104) in triplicate. Subsequently, 20 µL of the indicated dilution of concentrated S8-Ntox35 supernatant or control supernatant was added to each well, yielding a total volume of 100 µL per well. Plates were sealed with BreatheEasy gas-permeable film (Diversified Biotech BEM-1) and incubated at the indicated temperature with continuous orbital shaking in a BioTek Synergy HTX Multi-Mode Microplate Reader (Agilent). Optical density at 600 nm (OD_600_) and GFP-associated relative fluorescence units (RFU; excitation/emission 485/528 nm), when applicable, were recorded every 20 min throughout the incubation period.

### Bactericidal assay

Bactericidal activity of S8-Ntox35 was assessed using the same setup as the supernatant susceptibility assay, except viability was quantified by colony-forming units (CFU). Overnight cultures of the indicated bacterial strains were grown at 37°C with shaking and normalized to equivalent starting cell densities ( OD_600_= 0.25) in fresh BHI. Where indicated, cultures were treated with CCCP (5 µM final concentration) or an equivalent volume of DMSO as a vehicle control. CCCP or DMSO was added prior to aliquoting cultures into assay wells and was maintained throughout the intoxication period. Input viability (0 h) was determined immediately following supernatant addition. Samples were collected at 1 h and 2 h post-treatment, serially diluted in PBS, and plated on appropriate agar for CFU enumeration. Plates were incubated overnight at 37°C prior to counting. CFU counts were log₁₀-transformed prior to statistical analysis by one-way ANOVA with Dunnett’s multiple-comparison test.

### Contact competition

*L. monocytogenes* 10403S constitutively expressing GFP (*Lm*-GFP) and *S. aureus* strains were grown overnight in BHI broth at 37°C with agitation. Cultures were normalized to an optical density at 600 nm (OD_600_) of 0.1 in fresh BHI. Equal volumes of normalized cultures were mixed, and 5 µL of the mixture was spotted onto LB agar plates. Plates were incubated overnight at 37°C. Colonies were imaged using an iBright FL1500 imaging system (Thermo Fisher Scientific) to visualize GFP fluorescence. Colonies were excised with a sterile scalpel, resuspended in phosphate-buffered saline (PBS), serially diluted, and plated on BHI agar to enumerate total CFU and on BHI agar supplemented with streptomycin to selectively enumerate *L. monocytogenes* GFP. Competitive indices (CIs) were calculated as (*S. aureus*_final_/*L. monocytogenes*_final_)/(*S. aureus*_initial_/*L. monocytogenes*_initial_). CIs were log_10_ transformed prior to statistical analysis by one-way ANOVA with Dunnett’s multiple-comparison test. For qualitative imaging-based competition assays (Fig. S1C), cultures were instead normalized to an OD_600_ of 0.2 in PBS, mixed 1:1, spotted (1 µL) onto tryptic soy agar (TSA), and GFP fluorescence was imaged after 5 days.

### Suspension competition

To evaluate bacterial competition in liquid culture, *L. monocytogenes* constitutively expressing GFP (*Lm*-GFP) and *S. aureus* strains carrying the indicated pEPSA5 plasmids were grown overnight in brain heart infusion (BHI) broth at 37°C with agitation. Cultures were diluted to an OD_600_ of 0.1 in fresh BHI, and equal volumes were combined in wells of a sterile 96-well plate (GenClone 25-104) to a final volume of 100 µL. Plates were sealed with BreatheEasy gas-permeable film (Diversified Biotech) and incubated at 37°C with continuous orbital shaking in a BioTek Synergy HTX Multi-Mode Microplate Reader (Agilent). OD_600_ and GFP-associated relative fluorescence units (RFU) (excitation 485 nm and emission 528 nm) were measured every 20 min for 20 h. Each biological replicate was performed using independently grown overnight cultures.

### RNA extraction

An isolated colony of wild-type *L. monocytogenes* was inoculated from a BHI agar plate (1.5% [wt/vol] agar) supplemented with streptomycin (200 µg mL⁻¹) and grown overnight in 10 mL BHI broth containing streptomycin (200 µg mL⁻¹) at 37°C with shaking at 220 rpm. The overnight culture was divided into three 3 mL aliquots and treated with concentrated supernatant derived from *S. aureus* carrying either empty vector, wild-type S8-Ntox35, or S8-Ntox35^H205A^, added to a final concentration of 20% (vol/vol). Cultures were incubated for 1 h at 37°C with shaking (220 rpm).

Cells were harvested by centrifugation at 4,500 × *g* for 5 min at room temperature. Pellets were resuspended in 1 mL TRIzol reagent (Invitrogen) and transferred to bead-beating tubes (Benchmark Scientific, D1032-01). Mechanical lysis was performed by bead beating for 60 s, followed by 60 s on ice, repeated five times (total 5 min bead beating).

Following lysis, samples were briefly centrifuged to pellet beads, and the supernatant was transferred to Phase Lock Gel (5 Prime) tubes containing 500 µL chloroform. Samples were vigorously mixed by hand, incubated for 5 min at room temperature, and centrifuged at 13,000 × *g* for 15 min at 4°C. The upper aqueous phase was transferred into 500 µL isopropanol, gently inverted to mix, incubated for 10 min at room temperature, and precipitated overnight at −20°C.

RNA was pelleted by centrifugation at 13,000 × *g* for 15 min at 4°C, washed twice with 1 mL 70% (vol/vol) ethanol, and air-dried at room temperature for 30 min. Pellets were resuspended in 25 µL DEPC-treated water by incubation for 5 min at 65°C with vortexing every 30 s. Resuspended RNA was immediately chilled on ice and quantified using a NanoDrop spectrophotometer (Thermo Scientific).

### RNA urea PAGE

Analysis of RNA by acrylamide gel electrophoresis was performed using homemade 1.0 mm 12% acrylamide/7.5 M urea gels (29:1 acrylamide:bis-acrylamide) (National Diagnostics, EC-829). Prior to electrophoresis, 10 µg of total RNA per sample was denatured by heating to 65°C in the presence of UreaGel Loading Buffer (National Diagnostics) (95% [vol/vol] formamide, 18 mM EDTA, SDS, xylene cyanol, and bromophenol blue, pH 7.0). Gels were run using a mini-PROTEAN vertical electrophoresis system (Bio-Rad) at 200 V for 1.5 h. Total RNA was visualized by staining with SYBR Gold (1:10,000) for 15 min. Gels were imaged using a LI-COR Odyssey Fc.

### Experimental evolution

An overnight culture of Ntox35-sensitive *L. monocytogenes* constitutively expressing GFP (*Lm*-GFP) was back-diluted 1:100 into 1 mL of fresh BHI, and 100 µL of concentrated S8-Ntox35–containing supernatant was added. The culture was incubated overnight at 37°C with shaking. This procedure was repeated for three consecutive overnight passages. After the final passage, the evolved culture was assessed for Ntox35 susceptibility as described for the suspension competition. The evolved population was serially diluted in PBS and plated on BHI agar to obtain single colonies. Ten colonies were randomly selected for whole-genome sequencing, of which nine were successfully completed. Library preparation was performed using the Illumina DNA Prep Kit, and samples were sequenced on the iSeq 100 Sequencing System. FASTQ files obtained from sequencing were analyzed using the Variation Analysis tool available on the BV-BRC Resources platform (50, 51). Sequencing results are summarized in Table S1.

### Protein SDS-PAGE and western blotting

Concentrated supernatant samples were mixed with 4× Laemmli buffer containing β-mercaptoethanol to a final 1× concentration and boiled for 10 min at 95°C. Samples were centrifuged briefly and separated on 4%–20% Mini-PROTEAN TGX stain-free gels (Bio-Rad) alongside Precision Plus Protein All Blue prestained standards. Electrophoresis was performed at 100 V for 15 min, followed by 150 V for 35 min. Proteins were transferred to nitrocellulose membranes using a Trans-Blot Turbo transfer system (Bio-Rad).

Total protein was visualized using Revert Total Protein Stain (LI-COR) and imaged on an Odyssey Fc imaging system (LI-COR). Membranes were blocked for 1 h at room temperature in Intercept TBS blocking buffer (LI-COR) supplemented with human serum (1:500). Membranes were incubated overnight at 4°C with rabbit anti-Myc (Proteintech 16286-1-AP, 1:5,000) and mouse anti-HA (Abcam ab18181, 1:5,000) antibodies diluted in blocking buffer. Membranes were washed three times with TBS-T and incubated for 1 h at room temperature with IRDye 800CW goat anti-rabbit IgG and IRDye 680CW goat anti-mouse IgG secondary antibodies (LI-COR, 1:15,000). After additional washes, blots were imaged on an Odyssey CLx imaging system (LI-COR).

### Cytochrome *c* binding assay

A cytochrome *c* binding assay was performed to assess whether deletion of *dltA*, *mprF,* or *anrAB* led to an altered cell envelope charge. Overnight cultures of *L. monocytogenes* were grown in fresh BHI, and then, 1.5 mL of cells was collected by centrifugation at 1,500 × *g* and washed twice with 20 mM MOPS (3-[*N*-morpholino]propanesulfonic acid) buffer (pH 7). The washed cells were adjusted to an OD_600_ of 0.25 in the same buffer, and cytochrome *c* (Sigma-Aldrich, C3131) was added to a final concentration of 50 µg/mL. After a 10-min incubation at room temperature, the cell suspension was centrifuged at 16,000 × *g* for 5 min, and the supernatant was taken to measure absorbance at 410 nm. To determine a measure of relative cytochrome *c* binding, the absorbance of cytochrome *c* in the absence of cells was compared to the absorbance of cytochrome *c* with cells, using the following equation: % cytochrome *c* bound = 100 − [(OD_410_ with cells)/(OD_410_ without cells)] × 100.

### Growth curve analysis and lag time determination

Lag time was determined from bacterial growth curves by fitting optical density measurements to a parametric growth model. OD_600_ values were collected every 20 min. For each biological replicate, technical duplicates were averaged prior to curve fitting. Growth curves were fitted using a modified Baranyi-Roberts model, which estimates lag time (λ), maximum growth rate (µmax), and carrying capacity. Model fitting was performed in R using nonlinear regression, and lag time was extracted directly from the fitted parameters. Fits were visually inspected to ensure appropriate convergence and exclusion of aberrant curves. Lag times reported represent values derived from three averaged biological replicates.

## Data Availability

Sequencing data generated from experimentally evolved *L. monocytogenes* strains in this study have been deposited in the NCBI BioProject database under BioProject accession number PRJNA1466589. Associated Sequence Read Archive (SRA) accessions span SRR38620940–SRR38620949, and corresponding BioSample accessions span SAMN60145157–SAMN60145166.

## SUPPLEMENTAL MATERIAL

Structure S1 mbio.02804-25-s0001.txt PDB file related to Fig. 1B.

Structure S2 mbio.02804-25-s0002.txt PDB file related to Fig. 1C and Fig. S1B.

Structure S3 mbio.02804-25-s0003.txt PDB file related to Fig. 2A.

Structure S4 mbio.02804-25-s0004.txt PDB file related to Fig. S1A.

Structure S5 mbio.02804-25-s0005.txt PDB file related to Fig. S2A.

Structure S6 mbio.02804-25-s0006.txt PDB file related to Fig. S2B.

Structure S7 mbio.02804-25-s0007.txt PDB file related to Fig. S2C.

Supplemental Figures mbio.02804-25-s0008.pdf Fig. S1 to S7.

Structure File Legends mbio.02804-25-s0009.docx Description of each PDB file for AlphaFold structural predictions.

Supplemental Tables mbio.02804-25-s0010.xlsx Tables S1 to S7.

ASM does not own the copyrights to Supplemental Material that may be linked to, or accessed through, an article. The authors have granted ASM a non-exclusive, world-wide license to publish the Supplemental Material files. Please contact the corresponding author directly for reuse.
