# Supplementary material for: BetaH proteolysis unleashes an electrostatic-homing antibacterial polymorphic toxin
Source: mBio. 2026 Aug 5;17(9):e02804-25. doi: 10.1128/mbio.02804-25 (PMC13556192; doi:10.1128/mbio.02804-25)
Supplement: Supplementary file 1 [file mbio.02804-25-s0008.pdf]

Figure S1:

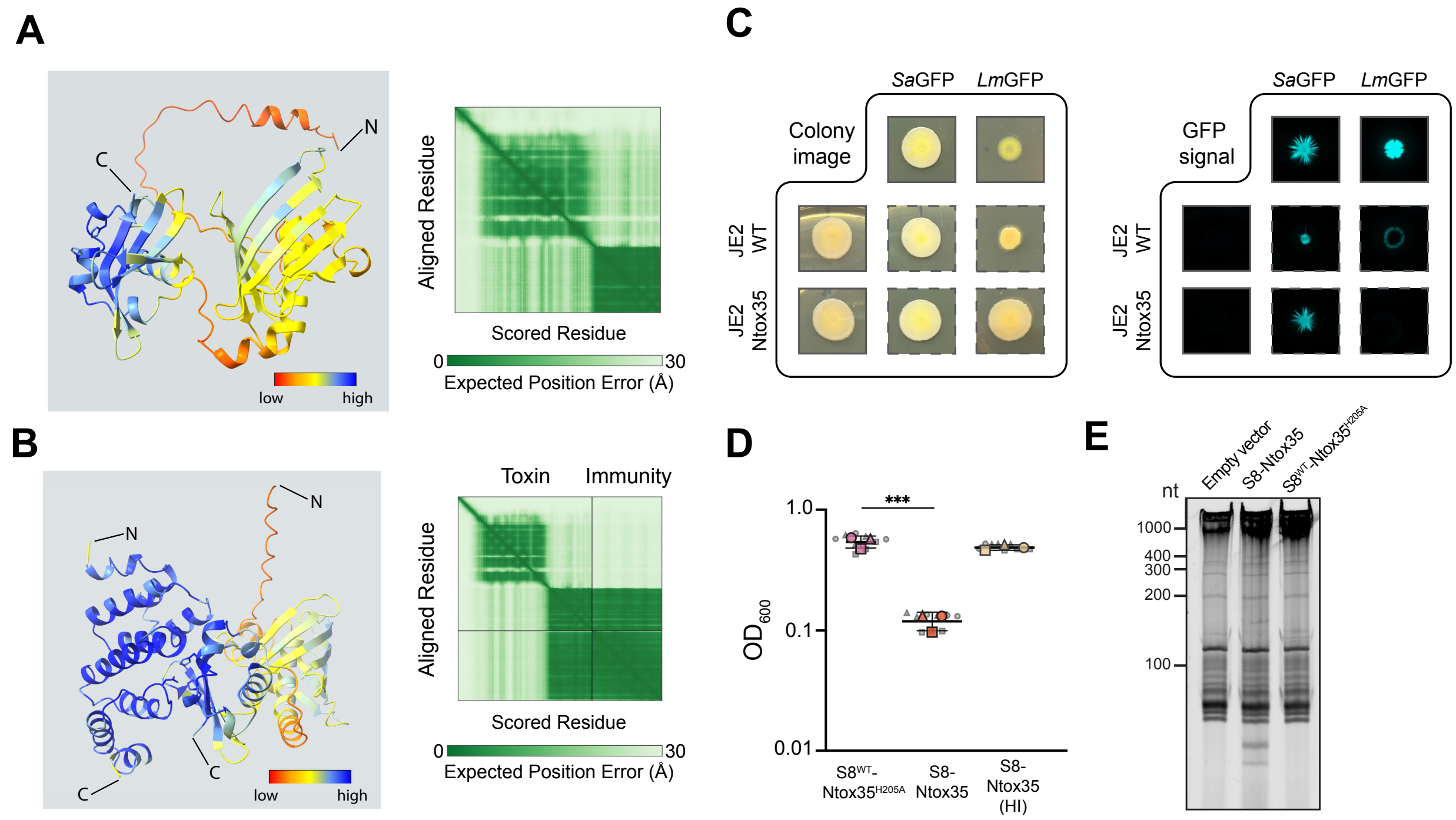

(A–B) AlphaFold3 structural models of (A) the BetaH-Ntox35 fusion protein (pTM = 0.45) and (B) the BetaH-Ntox35 complex with the cognate immunity protein TPR-Imm1 (ipTM = 0.91, pTM = 0.58). Structures are colored according to pLDDT confidence scores and the predicted aligned error (PAE) plot is shown.

(C) Imaging-based contact competition between GFP-expressing indicator strains and *S. aureus* strains encoding the indicated S8-Ntox35 loci. Assays were performed as described for contact-dependent competition, except GFP fluorescence was monitored on TSA plates without CFU enumeration. Representative images were acquired 5 d post-plating. Loss of GFP fluorescence indicates elimination of the indicator strain by contact-dependent intoxication.

(D) Growth of GFP-expressing *Listeria monocytogenes* (*Lm*-GFP), normalized to a starting density of approximately 5×10^5 CFU mL^{-1}, following exposure to the indicated *S. aureus*-derived supernatants (2.5% v/v). OD_{600} was recorded every 20 min during incubation, and values at 10 h—corresponding to stationary phase in untreated cultures—were used for comparisons. Data are presented as mean ± SD from three biological replicates (colored symbols, matched by shape), with corresponding technical replicates shown in gray. Asterisks indicate values significantly different from treatment with catalytic mutant supernatant ($S8^{WT}$-$Ntox35^{H205A}$) (one-way ANOVA with Dunnett's multiple-comparison test).

(E) Urea-PAGE analysis of total RNA extracted from wild-type *L. monocytogenes* following 1 h exposure to concentrated *S. aureus*-derived supernatants. RNA was resolved on a denaturing urea-polyacrylamide gel and stained with SYBR Gold. Distinct RNA cleavage products were observed following treatment with wild-type S8-Ntox35 supernatant but not with catalytic mutant or empty-vector control supernatants. Data are representative of three independent biological replicates.

Figure S2:

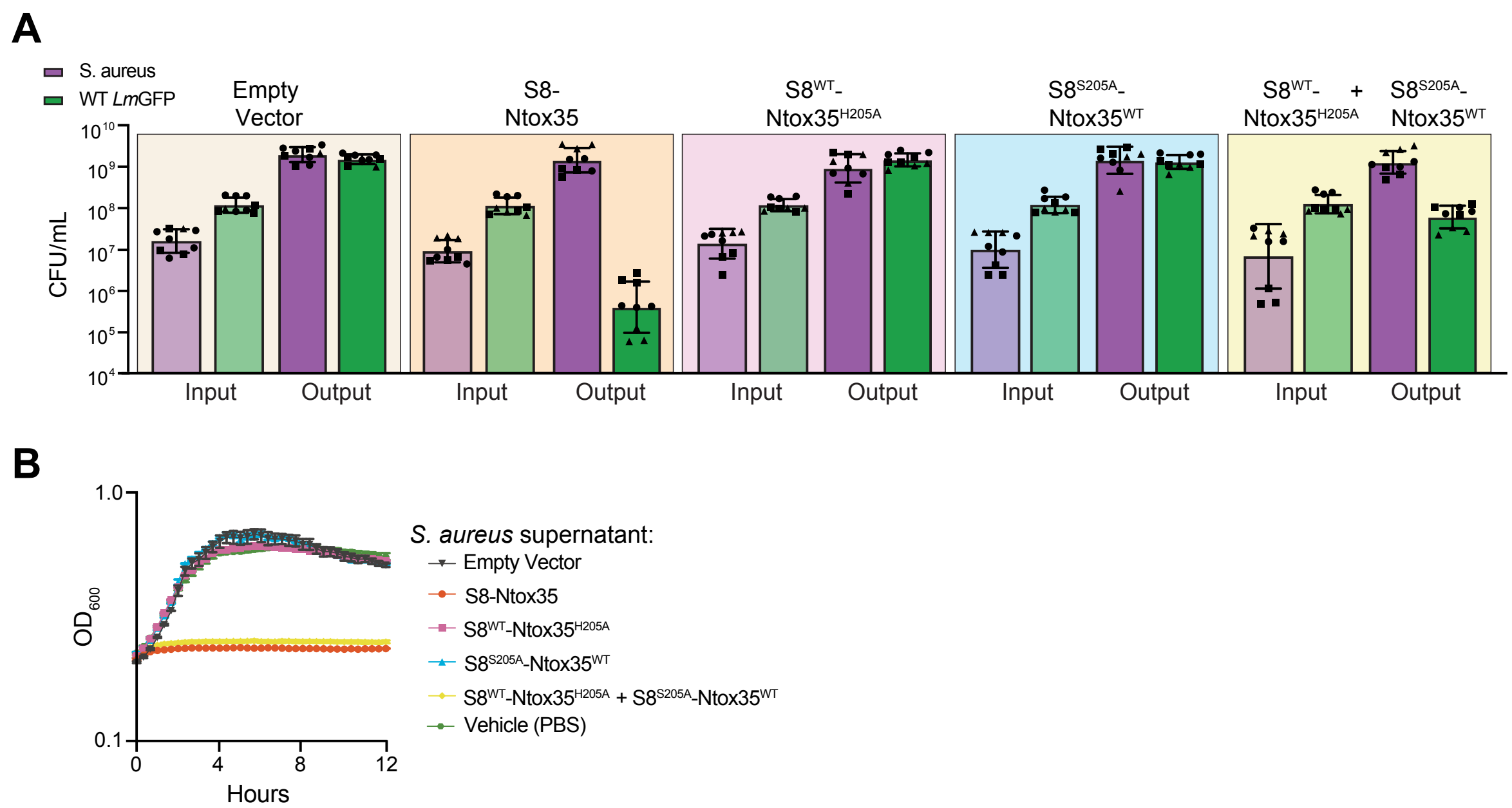

(A) Contact-dependent competition between *Listeria monocytogenes* GFP (*Lm*-GFP) and the indicated *S. aureus* strains was performed on solid agar. Colony-forming units (CFU) were enumerated on selective media for input and output samples. Data are presented as mean ± SD from n = 3 biological replicates (matched by shape).

(B) Growth of GFP-expressing *Listeria monocytogenes* (*Lm*-GFP) normalized to a starting OD_{600} of 0.25 and treated with indicated *S. aureus*-derived supernatants (2.5% v/v). Optical density at 600 nm (OD_{600}) was measured every 20 min in a microplate reader. Data points represent the mean ± SD of three technical replicates. The graph shown is representative of three independent biological experiments, each initiated from separate overnight cultures.

Figure S3:

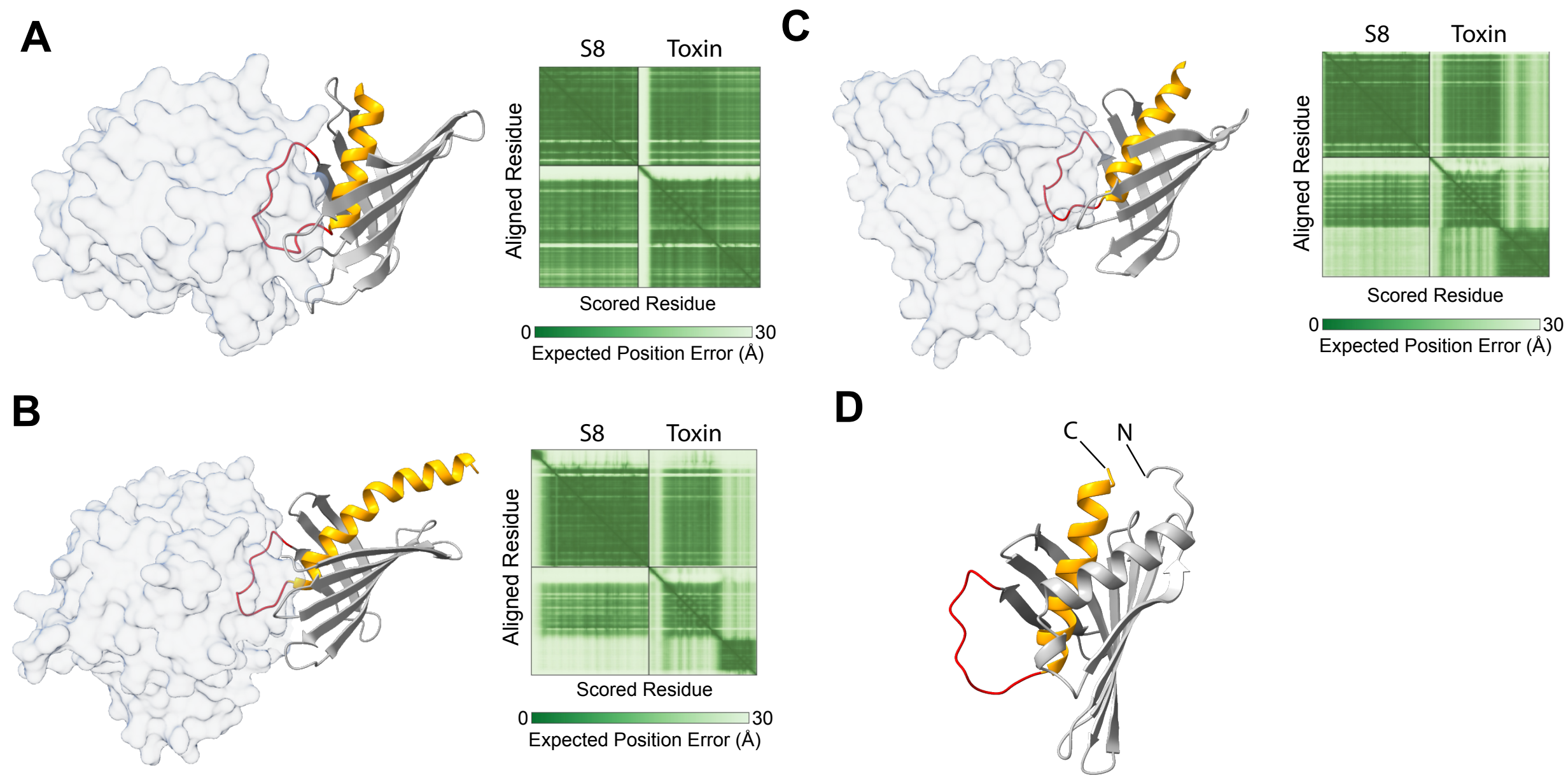

AlphaFold3 multimer models of cognate S8 peptidases (blue surface representation) and BetaH domains (β-sheet in gray and α-helix in tan ribbon representation) from **(A)** *Listeria monocytogenes* (ipTM = 0.84, pTM = 0.82), **(B)** *Anaerobacillus isosaccharinicus* (ipTM = 0.75, pTM = 0.68), and **(C)** *Pseudobacillus wudalianchiensis* (ipTM = 0.88, pTM = 0.74). In all models, the S8 peptidase active site is positioned adjacent to a flexible loop (red) separating the β-sheet and α-helical regions of the BetaH domain. The predicted aligned error (PAE) plot is shown. **(D)** Enlarged view of the interaction interface between the BetaH domain and toxin region from *P. wudalianchiensis*.

Figure S4:

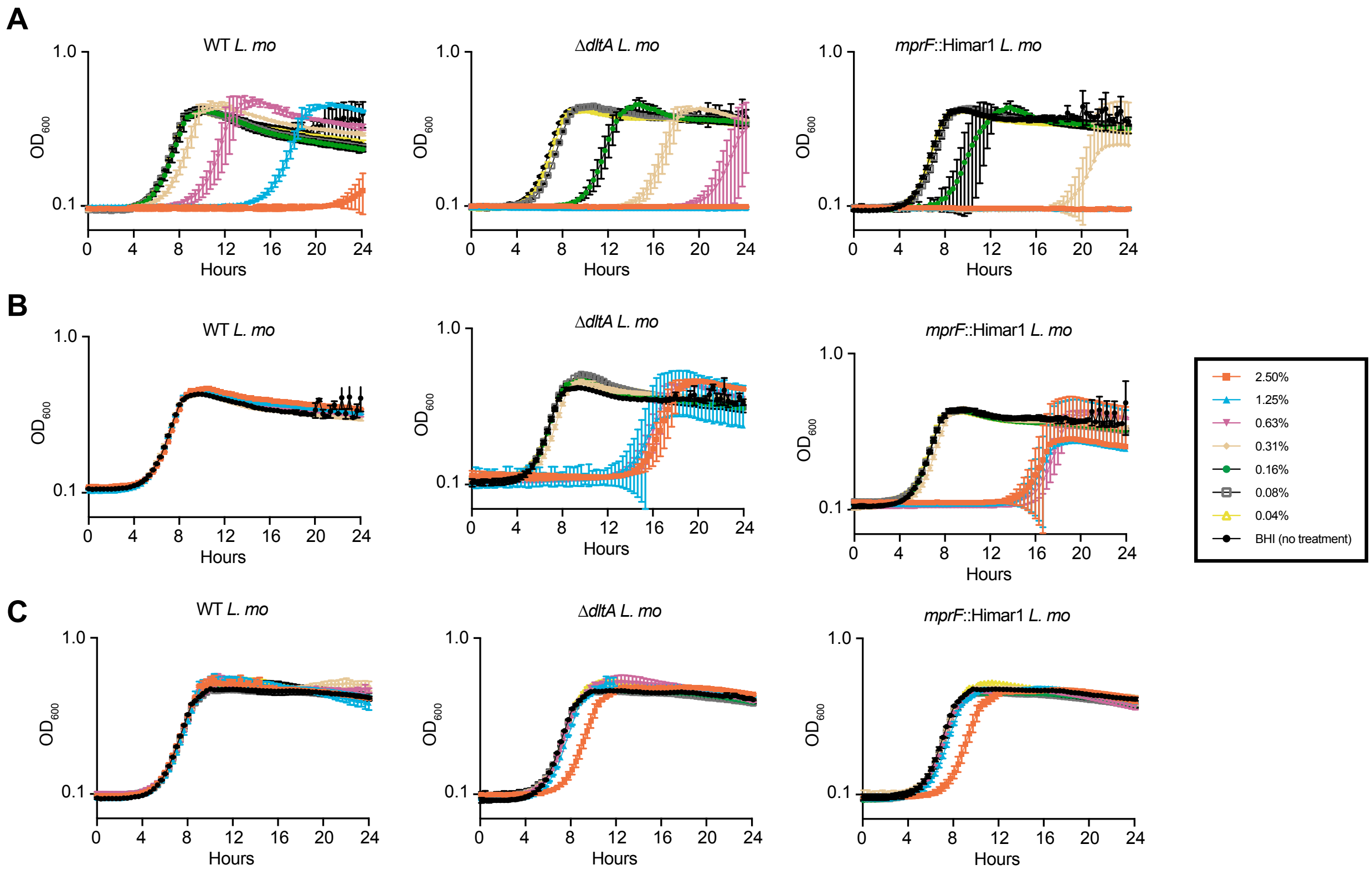

Growth of indicated *L. monocytogenes* strains, normalized to a starting density of approximately 5 × 10^5 CFU mL^{-1}, following treatment with titrated amounts of supernatant containing (**A**) wild-type S8–Ntox35, (**B**) unprocessed Ntox35 ($S8^{S205A}$-$Ntox35^{WT}$), or (**C**) catalytic mutant Ntox35 ($S8^{WT}$-$Ntox35^{H205A}$). Optical density at 600 nm (OD_{600}) was measured every 20 min during incubation in a microplate reader. Data are presented as mean ± SD from three technical replicates and are representative of three independent biological experiments initiated from separate overnight cultures.

Figure S5:

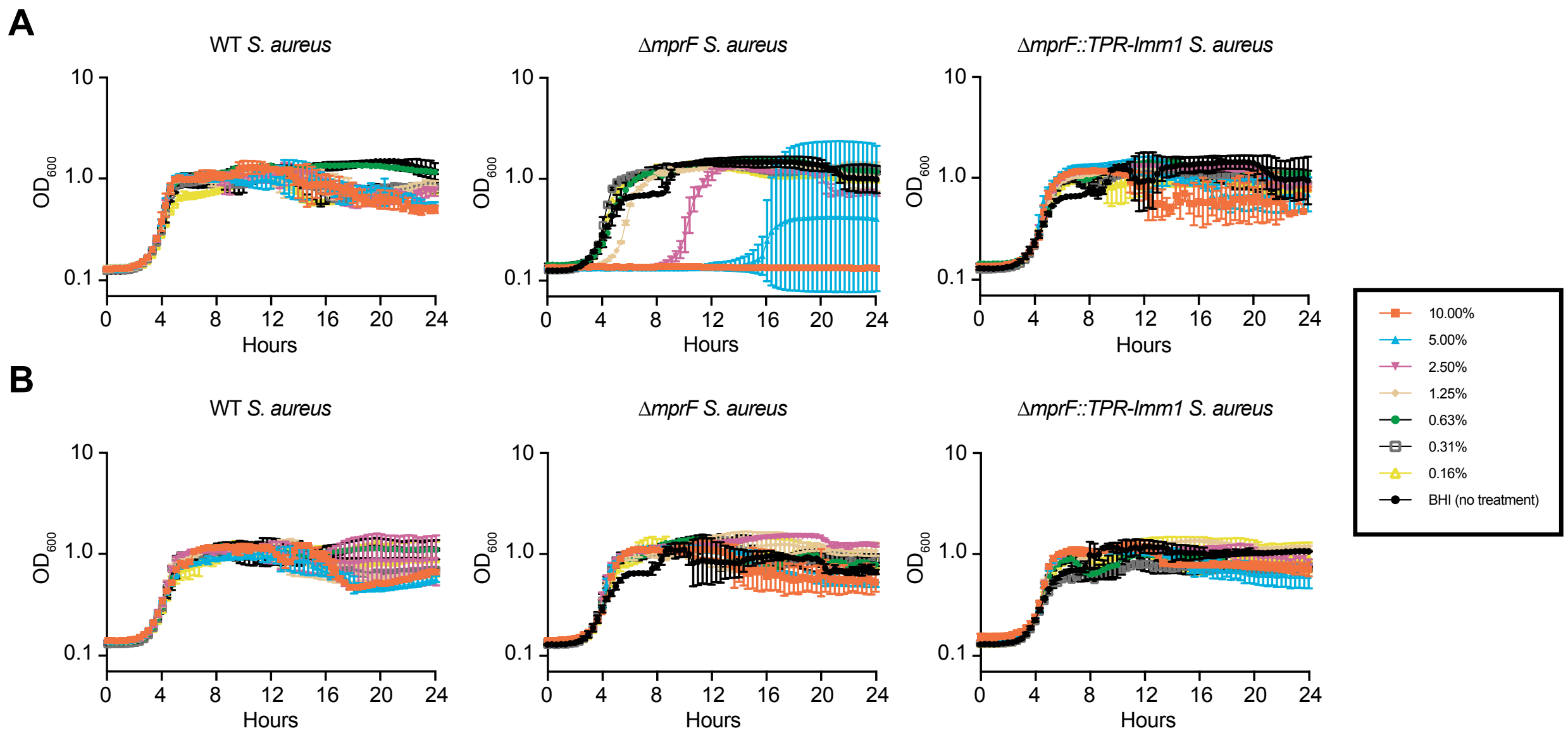

Growth of indicated *S. aureus* strains, normalized to a starting density of approximately 5 × 10^5 CFU mL^{-1}, following treatment with titrated supernatants containg (**A**) wild-type S8–Ntox35, or (**B**) catalytic mutant Ntox35 ($S8^{WT}$-$Ntox35^{H205A}$). Optical density at 600 nm (OD_{600}) was measured every 20 min during incubation in a microplate reader. Data are presented as mean ± SD from three technical replicates and are representative of three independent biological experiments initiated from separate overnight cultures.

Figure S6:

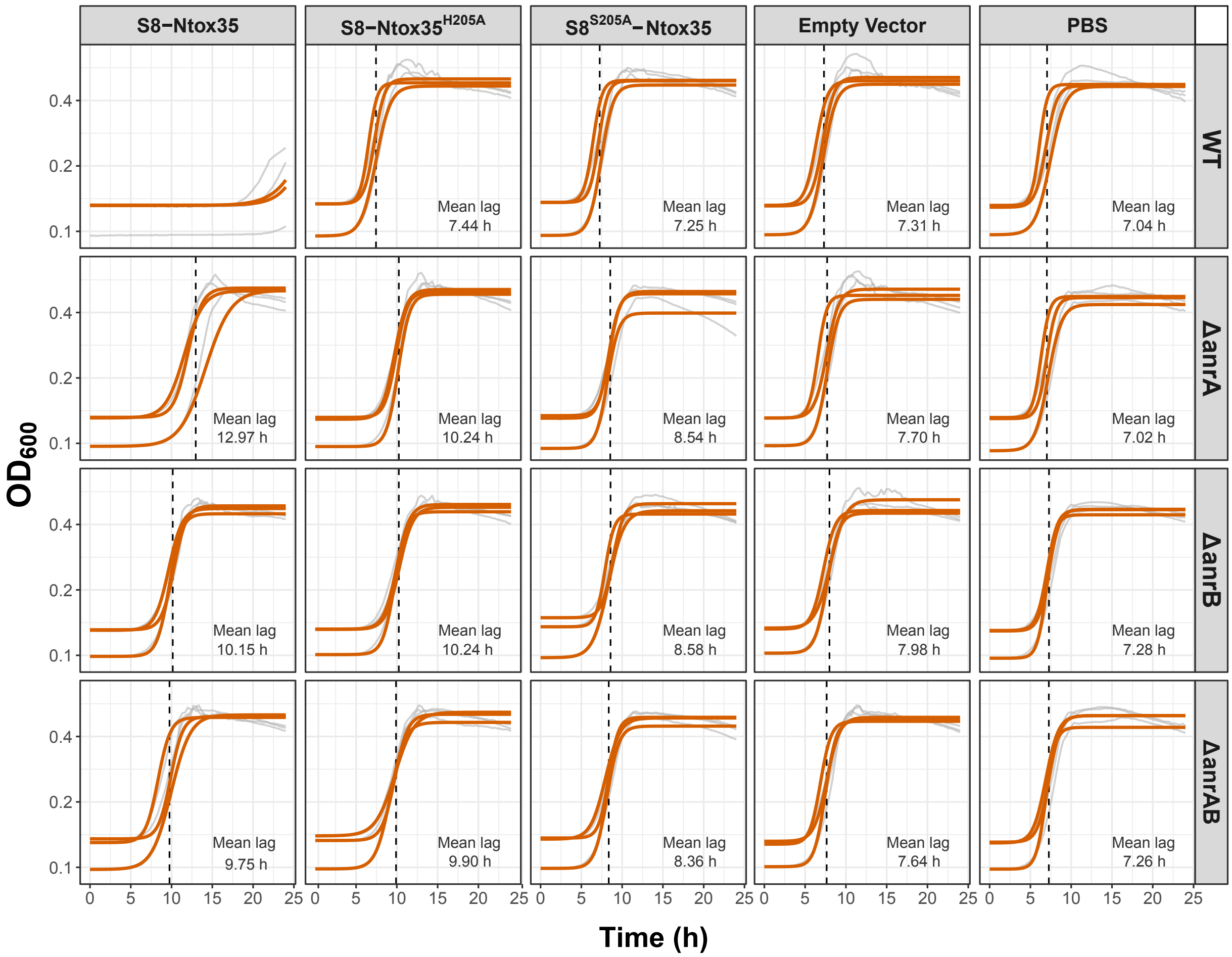

Growth of wild-type, Δ*anrA*, Δ*anrB*, or Δ*anrAB Listeria monocytogenes* strains normalized to a starting density of approximately 5 × 10^5 CFU mL^{-1} and treated with the indicated *S. aureus*-derived supernatants. Optical density at 600 nm (OD_{600}) was measured every 20 min in a microplate reader. Gray curves represent independent biological replicates (n=3 technical replicates) and orange curves represent Baranyi model fitted curves. Mean lag time calculate from fitted curves is displayed on each graph.

Figure S7:

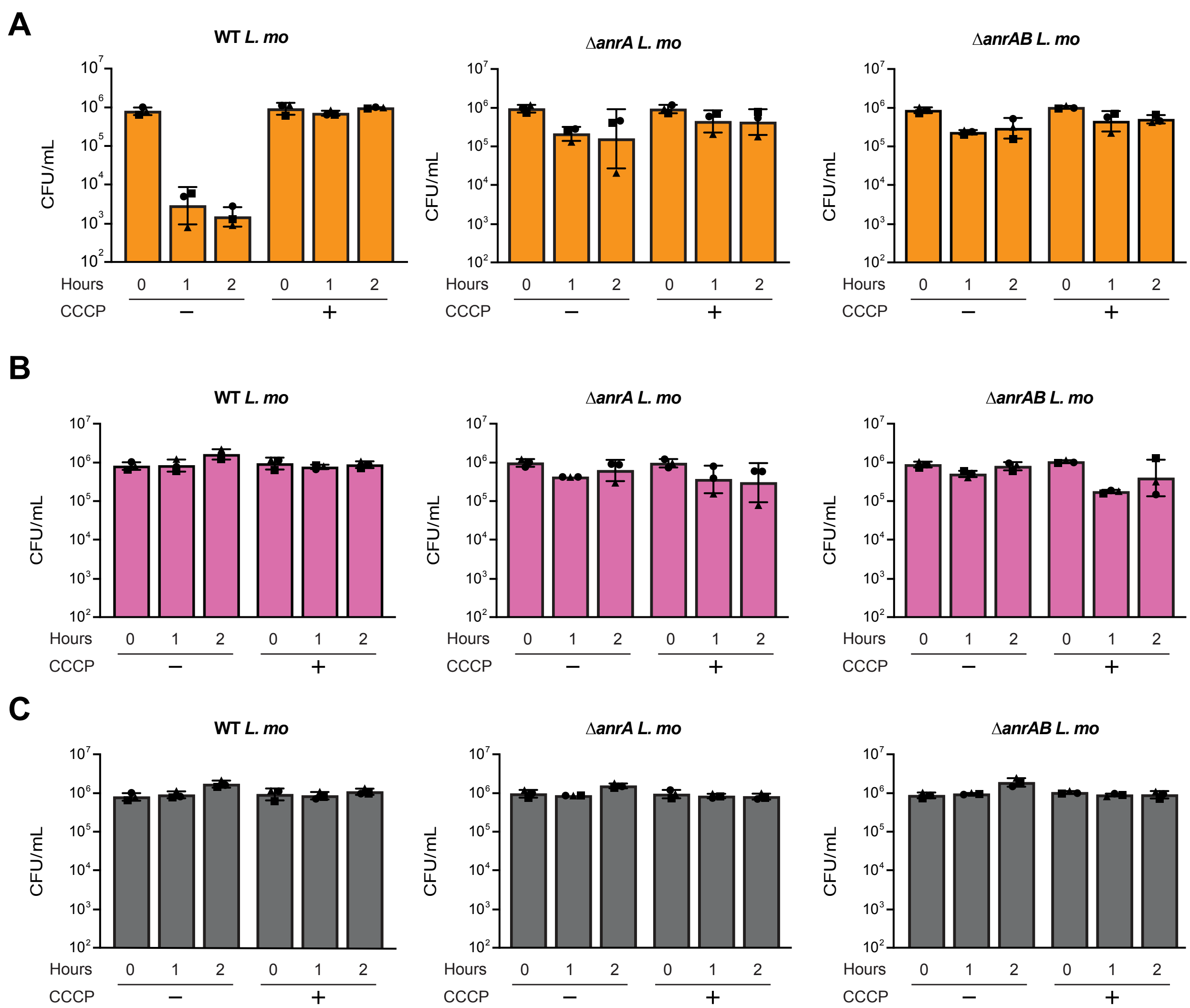

Survival of wild-type *L. monocytogenes*, Δ*anrA*, and Δ*anrAB* strains following exposure to *S. aureus*-derived supernatants containing **(A)** wild-type S8-Ntox35, **(B)** catalytic mutant Ntox35 ($S8^{WT}$-$Ntox35^{H205A}$), or **(C)** empty vector, in the presence or absence of the membrane-depolarizing agent CCCP. Viability was determined by CFU enumeration at 0, 1, and 2 h post-treatment.
