# Supplementary material for: BetaH proteolysis unleashes an electrostatic-homing antibacterial polymorphic toxin
Source: mBio. 2026 Aug 5;17(9):e02804-25. doi: 10.1128/mbio.02804-25 (PMC13556192; doi:10.1128/mbio.02804-25)
Supplement: Supplementary file 2 [file mbio.02804-25-s0009.docx]

Supplemental Material Structure File Legends

Supplemental Structure 1: Figure 1B PDB file of the top AlphaFold3 structural model of the Ntox35 toxin domain.

Supplemental Structure 2: Figure 1C and Supplemental Figure 1B PDB file of the top AlphaFold3 multimer structural model of the full length Ntox35 protein and the associated Immunity protein.

Supplemental Structure 3: Figure 2A PDB file of the top AlphaFold3 multimer structural model of the full length Ntox35 protein and the associated S8 Peptidase protein.

Supplemental Structure 4: Supplemental Figure S1A PDB file of the top AlphaFold3 structural model of the full length Ntox35 protein.

Supplemental Structure 5: Supplemental Figure S2A PDB file of the top AlphaFold3 multimer structural model of the *Listeria monocytogenes* Toxin protein and the associated S8 Peptidase protein.

Supplemental Structure 6: Supplemental Figure S2B PDB file of the top AlphaFold3 multimer structural model of the *Anaerobacillus isosaccharinicus* and the associated S8 Peptidase protein.

Supplemental Structure 7: Supplemental Figure S2C PDB file of the top AlphaFold3 multimer structural model of the *Pseudobacillus wudalianchiensis* and the associated S8 Peptidase protein.
